# Tools and tactics to define specificity of metabolic chemical reporters

**DOI:** 10.3389/fmolb.2023.1286690

**Published:** 2023-12-07

**Authors:** Mana Mohan Mukherjee, Michelle R. Bond, Lara K. Abramowitz, Devin Biesbrock, Carolyn C. Woodroofe, Eun Ju Kim, Rolf E. Swenson, John A. Hanover

**Affiliations:** ^1^ Laboratory of Cell and Molecular Biology, National Institute of Diabetes and Digestive and Kidney Diseases, National Institutes of Health, Bethesda, MD, United States; ^2^ Frederick National Laboratory for Cancer Research, National Cancer Institute, Fredrick, MD, United States; ^3^ Department of Chemistry Education, Daegu University, Gyeongsan-si, South Korea; ^4^ Chemistry and Synthesis Center, National Heart, Lung, and Blood Institute, National Institutes of Health, Bethesda, MD, United States

**Keywords:** bioorthogonal chemistry, metabolic chemical reporters, O- and N-glycans, hexosamine biosynthetic pathway, tools and tactics

## Abstract

Metabolic chemical reporters (MCRs) provide easily accessible means to study glycans in their native environments. However, because monosaccharide precursors are shared by many glycosylation pathways, selective incorporation has been difficult to attain. Here, a strategy for defining the selectivity and enzymatic incorporation of an MCR is presented. Performing β-elimination to interrogate *O*-linked sugars and using commercially available glycosidases and glycosyltransferase inhibitors, we probed the specificity of widely used azide (Ac_4_GalNAz) and alkyne (Ac_4_GalNAlk and Ac_4_GlcNAlk) sugar derivatives. Following the outlined strategy, we provide a semiquantitative assessment of the specific and non-specific incorporation of this bioorthogonal sugar (Ac_4_GalNAz) into numerous *N*- and *O*-linked glycosylation pathways. This approach should be generally applicable to other MCRs to define the extent of incorporation into the various glycan species.

## Introduction

**FIGURE 1:**
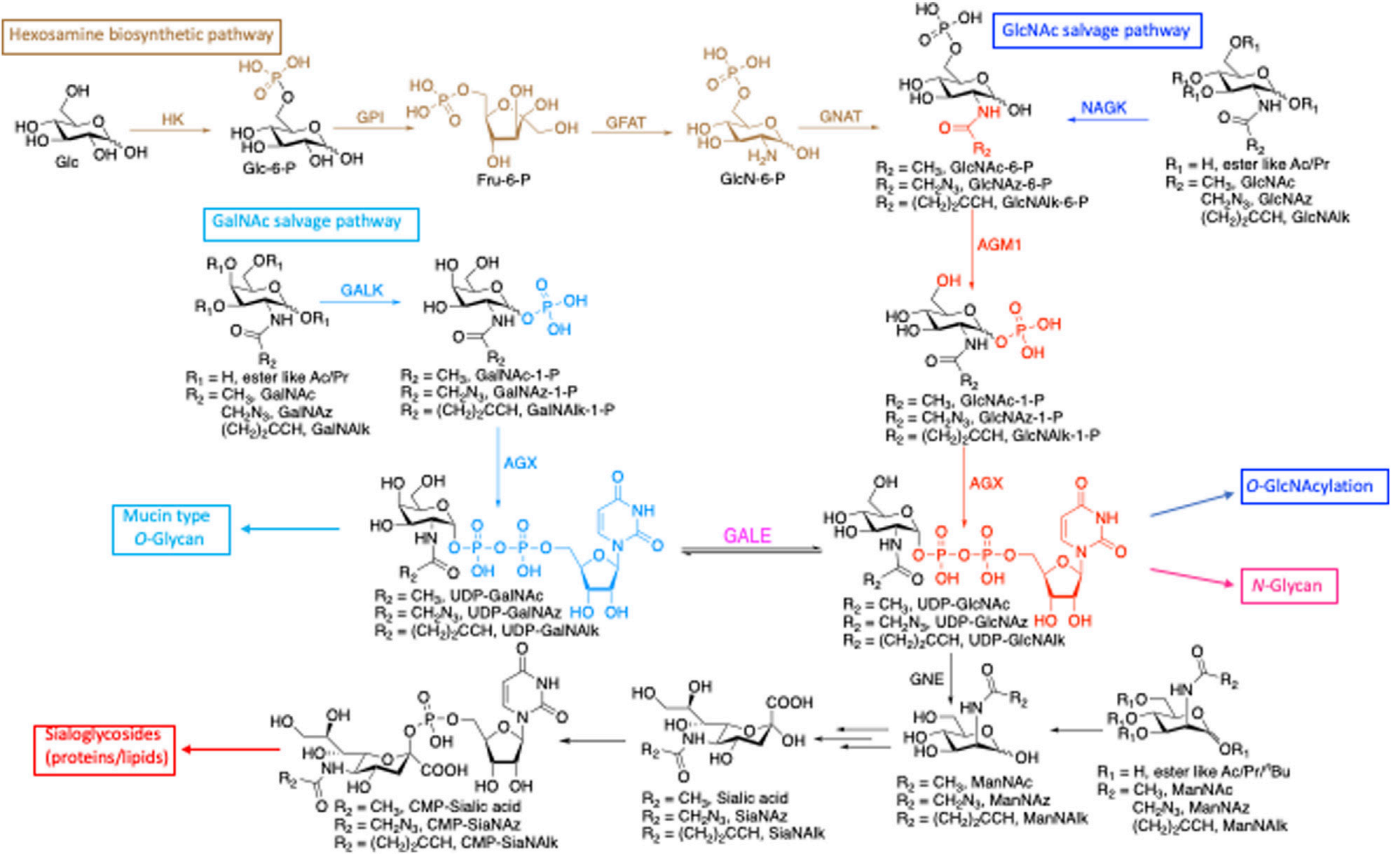
*De novo* biosynthetic and salvage pathways of GlcNAc and GalNAc metabolism highlight the shared precursors among glycoconjugates. HK, hexokinase; GPI, phosphoglucose isomerase; GFAT, glutamine:fructose-6-phosphate transaminase; GNAT, glucosamine-phosphate *N*-acetyltransferase; AGM1, GlcNAc phosphomutase; AGX, UDP-GlcNAc pyrophosphorylase; GALK, galactokinase; NAGK, *N*-acetylglucosamine kinase; GALE, UDP-glucose 4-epimerase, GNE, UDP-*N*-acetylglucosamine 2-epimerase/*N*-acetylmannosamine kinase.

Glycosylation is a versatile posttranslational modification (PTM) known to regulate many aspects of protein function (Rudd and Dwek, 1997; Bill et al., 1998; Konzman et al., 2020). There are three major forms of protein glycosylation that modify large numbers of protein substrates in mammalian cells. Proteins localized at the cell surface and secretory pathways can be modified with oligosaccharide structures, such as *N*-linked glycosylation (linked through asparagine) (Weerapana and Imperiali, 2006) or mucin *O*-linked glycosylation (linked through serine and threonine) (Hanisch, 2001). Cytoplasmic, nuclear, and mitochondrial proteins can also be modified with the single monosaccharide *N*-acetyl-glucosamine (*O*-GlcNAcylation through serine and threonine) (Love and Hanover, 2005). In addition to proteins, lipids and nucleic acids (NAs) can also be glycosylated (Flynn et al., 2021). These diverse glycosylations are derived from shared donor nucleotide sugars (Figure 1). UDP-GlcNAc, which is generated from glucose *de novo* through the hexosamine biosynthetic pathway (HBP) and from free GlcNAc via the salvage pathway, is a versatile precursor for several downstream substrates. These glycoconjugates include *O*-GlcNAcylation of intracellular proteins by *O*-GlcNAc transferase (OGT) (Haltiwanger et al., 1990; Haltiwanger et al., 1992; Hanover, 2001) and *N*-glycans by transferases in the Golgi (Stanley P et al., 2017; Stanley et al., 2022). UDP-GlcNAc can also be converted to its C4-epimer UDP-*N*-acetyl galactosamine (UDP-GalNAc) (Daude et al., 1995) by UDP-galactose-4′-epimerase (GALE) and incorporated into mucin-type glycans (Hang et al., 2003). A relatively minor pool of UDP-GlcNAc is converted to ManNAc and incorporated into sialic acid (Harms and Reutter, 1974), contributing to sialic acid containing glycoproteins (complex N-glycans) and glycolipids (ganglioside) (Du et al., 2009) (Figure 1).

Because glycosylation is not genetically encoded, tools to enable an intricate study of these modifications have lagged behind tools developed for templated molecules. Reutter and coworkers recognized the potential of exploiting sugar salvage pathways (Kayser et al., 1992; Keppler et al., 1995; Keppler et al., 2001) to assess glycosylation and synthesized a series of *N*-acetylmannosamine (ManNAc) derivatives bearing one, two, or three additional carbons on the *N*-acetyl position (Keppler et al., 1995). These analogs were successfully transformed into the corresponding sialic acid derivatives and installed on cell-surface glycoproteins. Soon after, the Bertozzi group started exploiting the promiscuities of these salvage pathways to allow the addition of small chemically reactive groups (Mahal et al., 1997; Saxon and Bertozzi, 2000; Bertozzi et al., 2001). They recognized that appending functional groups to glycoconjugates could facilitate covalent elaboration allowing for subsequent biochemical analysis. This approach relies on a two-step process. First is the metabolic incorporation of the unnatural sugar molecules, referred to as metabolic chemical reporters (MCRs), bearing biologically inert functional groups. Next, these functional groups must be coupled with selective and reactive partners to form covalent adducts. More specifically, small chemical handles such as azides or alkynes, which are tolerated at this position by the biosynthetic pathways and converted into nucleotide sugar donors, allow for monitoring of sugars *in vivo*. Glycosyltransferases can use these unnatural donors for the modification of proteins or lipids, and a subsequent bioorthogonal reaction step is then exploited to attach visualization or affinity tags for analysis.

Researchers continue to take advantage of an increasing selection of MCRs to label extra- and intracellular glycans with varying functional handles. Bioorthogonal tags, including ketones, azides, alkynes, 1,2,4,5-tetrazines, cyclopropenes, and diazirines, have been developed and utilized (Vocadlo et al., 2003; Zaro et al., 2011; Bateman et al., 2013; Chuh et al., 2014; Doll et al., 2016; Chuh et al., 2017). Furthermore, a number of MCRs are commercially available and can be used to label proteins with ‘click chemistry’, copper(I)-catalyzed azide–alkyne cycloaddition (CuAAC) (Rostovtsev et al., 2002; Tornøe et al., 2002; Prescher and Bertozzi, 2005; Patterson et al., 2014), a robust, selective, and bioorthogonal reaction whose components are readily available. With several bioorthogonal tags characterized, work towards tuning MCR structure for optimal biological availability and selectivity continues.

One limitation of the use of MCRs is that monosaccharide precursors are shared by many glycosylation pathways (Figure 1). To interrogate MCR selectivity, researchers have utilized many strategies after chemically labeling the bioorthogonal handle including immunofluorescence, immunoblotting, and proteomics. Each of these methods addresses different concerns and has both advantages and disadvantages. Immunofluorescence, for example, is a good method for monitoring the cellular localization of MCR incorporation but requires cells to be fixed. Differing compositions of organelles influence accessibility and staining (Schnell et al., 2012) and can create bias in interpretation. Immunoblots are excellent indicators of the level of incorporation in whole-cell lysates and can be expanded to monitor labeling of individual proteins when coupled with immunoprecipitation. However, localization and information on the type of glycan are lost unless the samples are fractionated or treated to distinguish between glycan types. Finally, proteomics has proven to be a high-throughput method to identify proteins labeled by unnatural MCRs; however, proteomic studies can be cost-prohibitive and difficult due to the lability of sugars.

**FIGURE 2:**
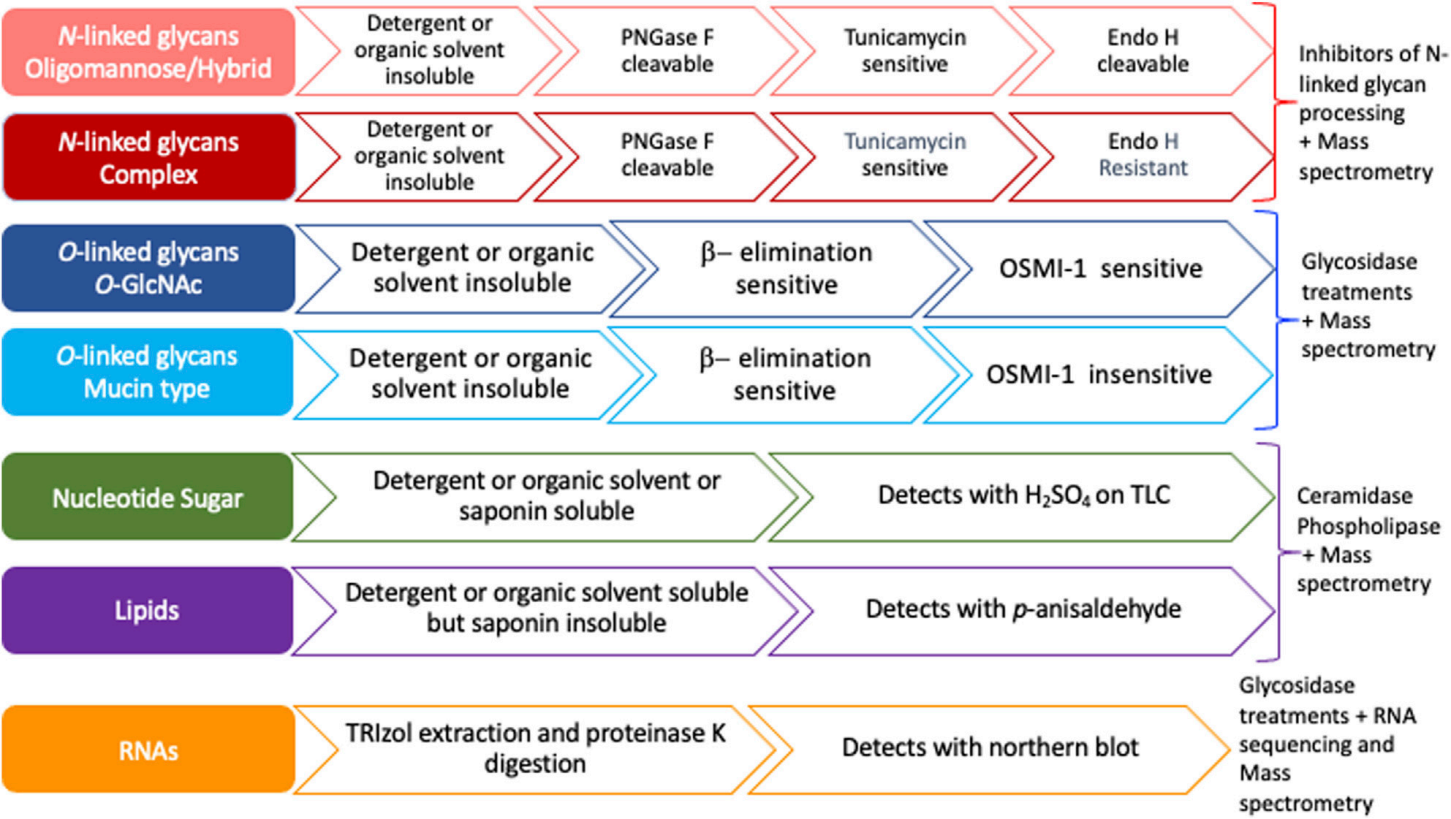
Flow chart to assess the specificity of metabolic chemical reporters. Nucleotide sugars and glycolipids are organic solvent (MeOH, acetone) and/or detergent (Triton X100) soluble and, hence, wash off during permeabilization or extraction using either of the two reagents. Both mucin type and intracellular *O*-GlcNAcylation are susceptible to an alkaline medium through β-elimination, whereas *N*-glycans or other non-enzymatic artificial *S*-modifications (linkage of cysteine residue to the C-3, C-4, or C-6 hydroxyl group in the sugar residue) are not. OSMI-1, an OGT inhibitor, reduces intracellular *O*-GlcNAcylation levels and has no direct impact on mucin-type glycan formation. PNGase F cleaves all three types of N-glycans and releases the oligosaccharide from the asparagine residue, whereas Endo H cleaves between the two *N*-acetyl-D-glucosamine residues in the chitobiose core for oligomannose and hybrid *N*-glycans but not complex *N*-glycans.

Previous studies have suggested that most MCRs are not selective for particular glycans and are instead incorporated into many glycoconjugates including intracellular and cell-surface glycoproteins, glycolipids, and glycoRNAs (Bond et al., 2010; Yu et al., 2012; Qin et al., 2018; Flynn et al., 2021). Furthermore, a compound resembling an alkynyl sugar but lacking a free anomeric hydroxy labeled acetyl groups by an unknown metabolic pathway (Zaro et al., 2014). Moreover, per-*O*-acetylated MCRs have been shown to non-enzymatically react with cysteine residues of intracellular proteins (Qin et al., 2018). Per-*O*-acetylated MCRs undergo base-promoted protein *S*-glyco modifications through an elimination–Michael addition reaction mechanism between free thiol residues present in cysteines and the ester functionalities present in the MCRs, particularly in the C-3 and C-4 positions of the carbohydrate scaffold (Qin et al., 2020). Here, we outline a strategy to interrogate the specificity and selectivity of an MCR and define the glycan class of incorporation (Figure 2). We suggest ways of utilizing the chemistry of proteins, lipids, and nucleic acids by exploiting extraction methods and solubilities to determine the type of molecule labeled (Figure 2). We profile the commercially available bioorthogonal sugars: Ac_4_GalNAz, Ac_4_GalNAlk, and Ac_4_GlcNAlk, as an example. These precursors were chosen due to their known incorporation onto proteins and published glycomic studies, allowing us to confirm the efficacy of our strategy (Zaro et al., 2014; Woo et al., 2015). Using both chemical and genetic tools, we monitored the compounds’ incorporation into *O*- and *N*-linked glycans. Although other studies have investigated the specificity and selectivity of MCRs on an individual basis, we believe that the systematic strategy presented here (Figure 2) will provide a framework for other laboratories to easily adopt in order to better understand the incorporation of MCRs and the nuances of *in vivo* glycan labeling.

## Results

Bioorthogonal sugars, including those labeled by azides and alkynes, are important tools for identifying glycosylated proteins in both tissue culture cells and whole organisms (Vocadlo et al., 2003; Boyce et al., 2011; Zaro et al., 2011). Although the use of MCRs has become widespread, there is a lack of an accepted standard systematic approach to test the specificity and selectivity of any given MCR. Here, we provide a methodological framework for researchers to test the selectivity and specificity of MCRs (Figure 2 and Supplementary Figure S1). Our workflow proposes that after the treatment of cells with the MCR of interest, the investigator first determine whether the labeling can be detected after treatment with various kinds of detergent (non-ionic and cholesterol-specific) with or without formaldehyde/paraformaldehyde fixation (Fox et al., 1985; Goldenthal et al., 1985). If the signal remains after fixation and detergent treatment, the MCR likely modified a protein and will require further interrogation to determine the linkage. Of course, the nature of the bioorthogonal tag must be taken into consideration to ensure no “fixable” groups could interfere with the interpretation. If the MCR is incorporated into proteins, linkage analysis can be performed. This can be carried out by the assessment of base-lability, cleavage with specific glycosidases such as PNGase F and Endo H, and inhibitors of glycosyltransferases: tunicamycin for *N*-linkage and OSMI-1 for *O*-GlcNAc. If the signal is lost after detergent/organic solvent extraction, or post *p*-formaldehyde (PFA) fixation and permeabilization with Triton X100 or saponin (Goldenthal et al., 1985; Schwarz and Futerman, 1997), the MCR likely modified lipids or remained as a nucleotide sugar. If glycolipids are indicated, a lipid fraction can be prepared by Folch extraction (Folch et al., 1957) and analysis by HPLC or TLC for the various classes of glycolipids differing in their polarity. On TLC, detection of the extracted compounds can be performed with H_2_SO_4_ or *p-*anisaldehyde. If extraction of nucleotide sugars is required, several methods of extraction can be performed including perchloric acid extraction (Kochanowski et al., 2006) and methanol extraction (Dietmair et al., 2010). Alternatively, if the MCR is incorporated into a glycoRNA, it can be extracted with TRIzol in the presence of proteinase K digestion and detected by Northern blot (Figure 2, Supplementary Figure S1).

To demonstrate the utility of the proposed strategy, we used well-studied and commercially available MCRs that are known to modify proteins and have available glycomics. Here, we treated HeLa cells with either Ac_4_GalNAz (main figures) or an alkyne derivative Ac_4_GalNAlk or Ac_4_GlcNAlk (Supplementary Figure). A click reaction was performed on the cell lysates to label azide/alkyne moieties with the respective counter TAMRA alkyne/azide for visualization. We followed the outlined strategy (Figure 2 and Supplementary Figure S1) to interrogate the following: I) glycan linkage by exploiting base-lability and commercially available glycosidases and II) enzymatic incorporation by chemical or genetic inhibition of specific glycosyltransferases.

### Determining the proportion of glycans labeled by our MCRs that are *O*-linked

**FIGURE 3:**
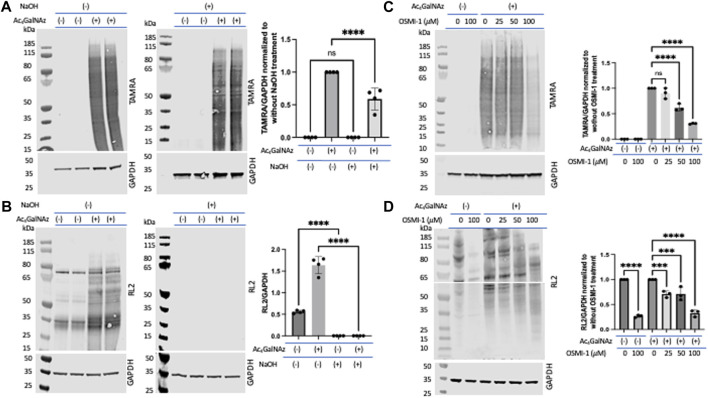
Portion of *O*-linked glycans labeled by Ac_4_GalNAz. On-blot β-elimination reduced Ac_4_GalNAz labeling. **(A)** Representative blot showing the Ac_4_GalNAz signal with or without NaOH treatment, and graphical quantitation of TAMRA labeling normalized to GAPDH of all analyzed replicates (TAMRA labeling, N = 4, an ordinary one-way ANOVA test shows ****p < 0.0001, ns = not significant, lanes 1 and 2 are replicates of DMSO-treated cell lysates, and lanes 3 and 4 are replicates of Ac_4_GAlNAz-treated cell lysates). **(B)** Representative blot showing endogenous *O*-GlcNAc signals (RL2) with or without NaOH treatment and graphical quantitation of RL2 normalized to GAPDH for all analyzed replicates (RL2 signals, N = 4, an ordinary one-way ANOVA test shows ****p < 0.0001, lanes 1 and 2 are replicates of DMSO-treated cell lysates, and lanes 3 and 4 are replicates of Ac_4_GAlNAz-treated cell lysates). **(C)** Representative blot showing that the OGT inhibitor OSMI-1 decreases Ac_4_GalNAz labeling and graphical quantitation of all replicates (TAMRA signals, N = 3, an ordinary one-way ANOVA test shows ****p < 0.0001, ns = not significant). **(D)** Representative blot showing diminished endogenous *O*-GlcNAc (RL2) detection in a concentration-dependent manner. The blot was cut at 55 kDa to allow analysis of indicated antibodies, and graphical quantitation of all replicates (N = 3, an ordinary one-way ANOVA test shows ****p < 0.0001 and ***p = 0.0008 or 0.0009).

β-Elimination is a technique known to cleave *O*-linked glycans while leaving *N*-linked glycans intact (Fukuda, 1994; Zachara et al., 2004; Fahie et al., 2021). We note that *O*-linked glycans are sensitive to β-elimination, and we would expect that *O*-linked GlcNAz would also be base-labile. To assess the level of incorporation of Ac_4_GalNAz in treated HeLa cells, a click reaction was performed on cell lysates to label the azide moiety with a TAMRA alkyne. Lysates were run out on a denaturing gel and transferred to nitrocellulose. The blot was either incubated in water or 55 mM NaOH for 24 h at 40 °C. An anti-TAMRA antibody was used to assess the levels of the MCR after β-elimination (Figure 3A). In comparison to the water-treated blots, the signal after β-elimination decreased by approximately 40% (Figure 3A). We confirmed the β-elimination conditions through an assessment of endogenous *O*-GlcNAc (using the RL2 antibody) and witnessed a complete loss of signal for *O*-GlcNAc (Figure 3B). These data suggest that only a portion of the azide-derived signal was due to GalNAz-modified *O*-linked glycans. A similar observation was detected with corresponding alkyne derivatives Ac_4_GalNAlk or Ac_4_GlcNAlk. When assessing the alkyne derivatives, we also treated cells with the OGA inhibitor thiamet G in hopes to accumulate an increased level of incorporation into *O*-GlcNAc (Supplementary Figure S2A); however, this had no impact on the amount of TAMRA signal with or without base treatment.

Next, we assessed the enzymatic incorporation of the azide derivative into intracellular *O*-GlcNAc. Inhibition of glycosyltransferases is an important component in assessing MCRs as non-enzymatic labeling has been described (Qin et al., 2018) and is a potential artifact when using MCRs. To do this, we treated cells with the OGT inhibitor OSMI-1 prior to treatment with Ac_4_GalNAz. We found that this treatment substantially decreased the amount of MCR labeling (Figure 3C). OGT inhibition was confirmed through an assessment of endogenous *O*-GlcNAcylation (detected with the RL2 antibody) (Figure 3D). Therefore, the majority of the MCRs that incorporated into *O*-linked glycans were mediated by OGT.

Alternatively, another approach to assess enzymatic incorporation would be to genetically knock down the glycosyltransferase using siRNAs. This is ideal if there is not a commercially available inhibitor to the glycosyltransferase of interest. As an example, we used siOGT to knockdown OGT in cells and then treated with the alkyne Ac_4_GlcNAlk (Supplementary Figure S2B). *O*-GlcNAc and OGT levels were reduced significantly with siOGT. However, GlcNAlk labeling, as determined using an anti-TAMRA antibody, increased after OGT knockdown (Supplementary Figure S2B). These data indicated that the decrease in TAMRA signal after β-elimination with the alkyne derivatives was unlikely to be due to enzymatic incorporation into *O*-GlcNAc (Supplementary Figure S2A).

It is possible that endogenous glycanases will not recognize the modified sugar, making targeting the glycanase to assess incorporation difficult. Here, we give an example of mutagenizing the glycanase so that it can recognize the MCR. Although the azide derivative has been shown to be recognized and removed by OGA (Boyce et al., 2011), the alkyne derivatives have not. Here, we used an *in vitro* GlcNAcase assay utilizing coumarin derivatives described in Materials and methods. We leveraged the recombinant bacterial OGA (Supplementary Figure S3B), BtGH84, for these assays (Vocadlo, 2012). We found that while wildtype (WT) BtGH84 did not recognize GlcNAlk, one of the mutants, the C277A mutant, was able to cleave this modification (Supplementary Figure S3B). Next, we incubated cell lysates from cells treated with the alkyne derivatives with either WT BtGH84, the C277A mutant, or nothing and found that while the *O*-GlcNAc signal disappeared with both the wildtype and mutant glycanases (as detected by RL2), the alkyne signal (detected with the anti-TAMRA antibody) remained (Supplementary Figure S3C). Thus, these data support the siOGT analysis indicating limited incorporation into the *O*-GlcNAc pathway.

### Portion of glycans labeled by our MCRs that are *N*-linked

**FIGURE 4:**
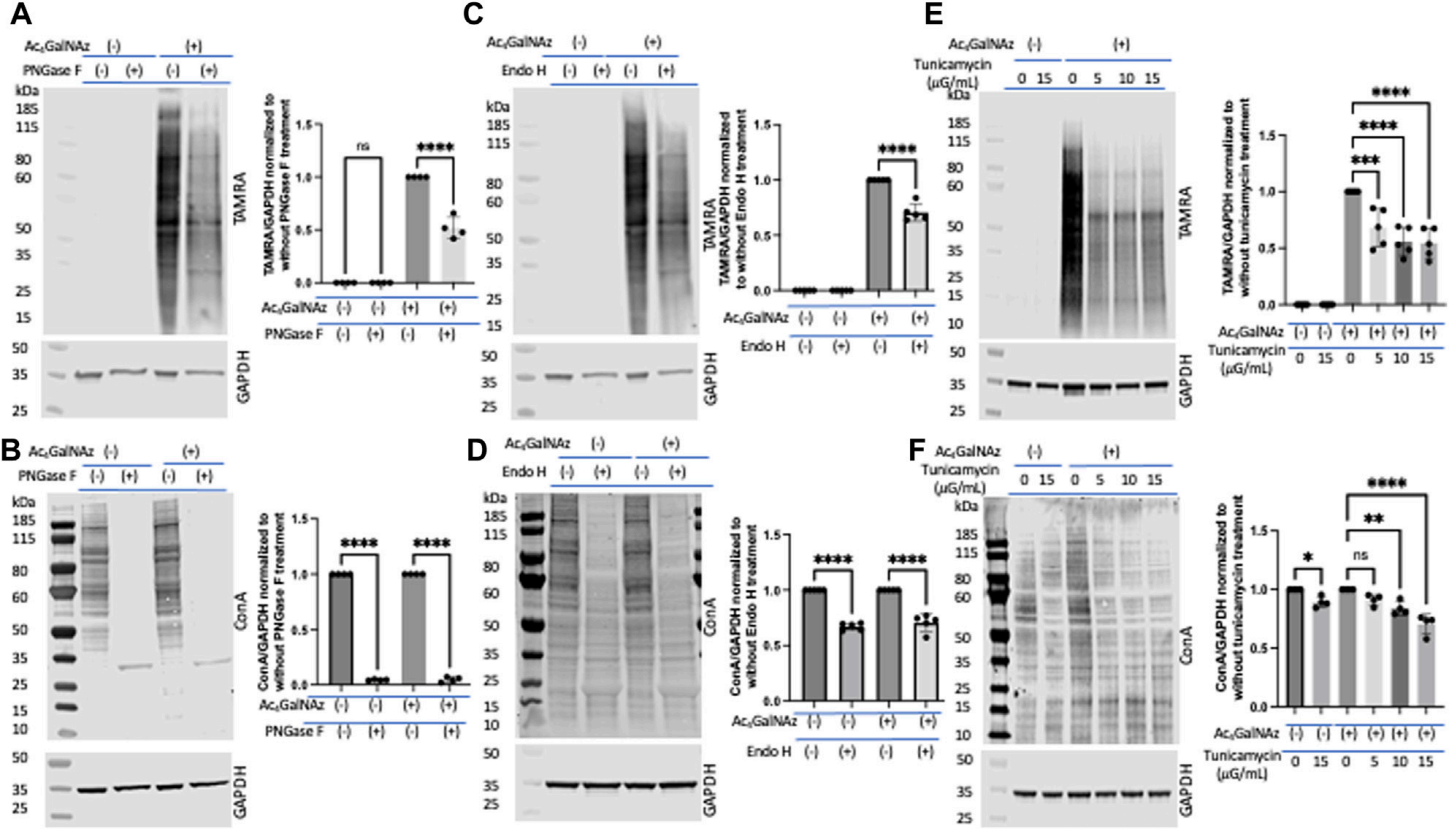
Portion of *N*-linked glycans labeled by Ac_4_GalNAz. **(A)** Representative blot showing PNGase F treatment significantly diminished Ac_4_GalNAz labeling with graphical quantitation of all replicates (TAMRA signal, N = 4, an ordinary one-way ANOVA test shows ****p < 0.0001, ns = not significant). **(B)** Representative blot showing that PNGase F treatment removed endogenous lectin labeling and graphical quantitation of all replicates (Con A signal, N = 4, an ordinary one-way ANOVA test shows ****p < 0.0001). **(C)** Representative blot showing that Endo H treatment decreased Ac_4_GalNAz labeling and graphical quantitation of all replicates (TAMRA signal, N = 5, An ordinary one-way ANOVA test shows ****p < 0.0001). **(D)** Representative blot showing that Endo H treatment decreases the endogenous lectin labeling (Con A signal, N = 5, an ordinary one-way ANOVA test shows ****p < 0.0001) and graphical quantitation of all replicates. **(E)** Representative blot showing *N*-glycan biosynthesis inhibitor tunicamycin reduced Ac_4_GalNAz labeling and graphical quantitation of all replicates (TAMRA signal, N = 5, an ordinary one-way ANOVA test shows ****p < 0.0001 and ***p = 0.0001). **(F)** Representative blot showing diminished endogenous lectin labeling (Con A) in a concentration-dependent manner and graphical quantitation of all replicates (N = 4, an ordinary one-way ANOVA test shows ****p < 0.0001, **p = 0.0011, *p = 0.0283, and ns = not significant). For these studies, the cells were trypsinized for removal from tissue culture plate.

Next, following the proposed strategy, we set out to interrogate incorporation into *N*-linked glycoconjugates. We expected that a significant portion of the signal from the MCR was due to *N*-linked glycans as this has been shown by others (Hang et al., 2003; Hsu et al., 2007; Boyce et al., 2011; Marotta et al., 2012). To probe *N*-linked incorporation, we took advantage of commonly used enzymatic and inhibitor techniques. Lysates from HeLa cells separately treated with MCRs and DMSO were subjected to PNGase F (Maley et al., 1989; Zachara et al., 2004; Darabedian et al., 2020) and Endo H (Freeze and Kranz, 2010) digestion, two well-characterized glycosidases. This experiment required that the enzymes tolerate the varied sugar structure as has been previously suggested by the cleavage of Ac_4_GlcNAz (Breidenbach et al., 2010). PNGase F is an amidase which cleaves between the innermost GlcNAc and asparagine residues of complex, hybrid, and high-mannose oligosaccharides. Lysates from Ac_4_GalNAz- or DMSO-treated HeLa cells were treated with PNGase F followed by a click reaction to add a TAMRA alkyne. Samples were run out on a gel, transferred to nitrocellulose, and an anti-TAMRA antibody was used to assess levels of MCR (Figure 4A) labeling. There was approximately a 50% decrease in TAMRA signal after PNGase F treatment. To confirm that the enzymatic treatment worked, we assessed the binding of lectin concanavalin A (Con A) which binds terminal mannosyl and glucosyl groups. We found a significant reduction in Con A binding after PNGase F treatment as anticipated (Figure 4B). Endo H hydrolyzes the bond between the two GlcNAc units that comprise the chitobiose core of the oligomannose and the hybrid-type *N*-glycan structures but not complex-type glycans (Freeze and Kranz, 2010). Thus, Endo H treatment should account for the disappearance of only the oligomannose and hybrid *N*-glycans which incorporate GlcNAz at any position except the first one of the chitobiose core attached to the asparagine residue. Endo H treatment resulted in partial depletion of the TAMRA-labeled signal (Figure 4C), akin to PNGase F (Figure 4A) treatment, and significant loss of Con A signal on Western blot (Figure 4D). However, we found no change in the TAMRA signal with the alkyne derivatives (Supplementary Figure S4). These data suggest that a significant portion of the azide-modified glycans were *N*-linked (all three types of *N*-glycans, namely, oligomannose, hybrid, and complex), whereas the alkynes were not. It is also possible that the addition of the alkyne to the *N*-acetyl position precludes PNGAse F or Endo H from cleaving the modified species (Plummer and Tarentino, 1991; Darabedian et al., 2020). Because trypsinization can remove a portion of cell-surface sugars (Batt et al., 2017), we repeated the PNGase F analysis on Ac_4_GalNAz-treated cells that had been scraped for removal from the tissue culture plate (Supplementary Figure S5A,B) and found comparable results to those presented in Figures 4A,B when cells were trypsinized.

Next, to assess enzymatic incorporation of the azide sugar into *N*-glycans, cells were treated with the well-studied *N*-glycan biosynthesis inhibitor tunicamycin (Elbein, 1987). With increasing tunicamycin concentration, both the azide signal (detected with anti-TAMRA antibody) (Figure 4E) and endogenous *N*-glycans (detected with Con A) (Figure 4F) decreased significantly. Together, these experiments suggest that a significant quantity of the azide-derivative MCR was enzymatically incorporated into *N*-glycans.

### A relatively smaller portion of the Ac_4_GlcNAz labeled beyond *N*- and *O*-glycans

**FIGURE 5:**
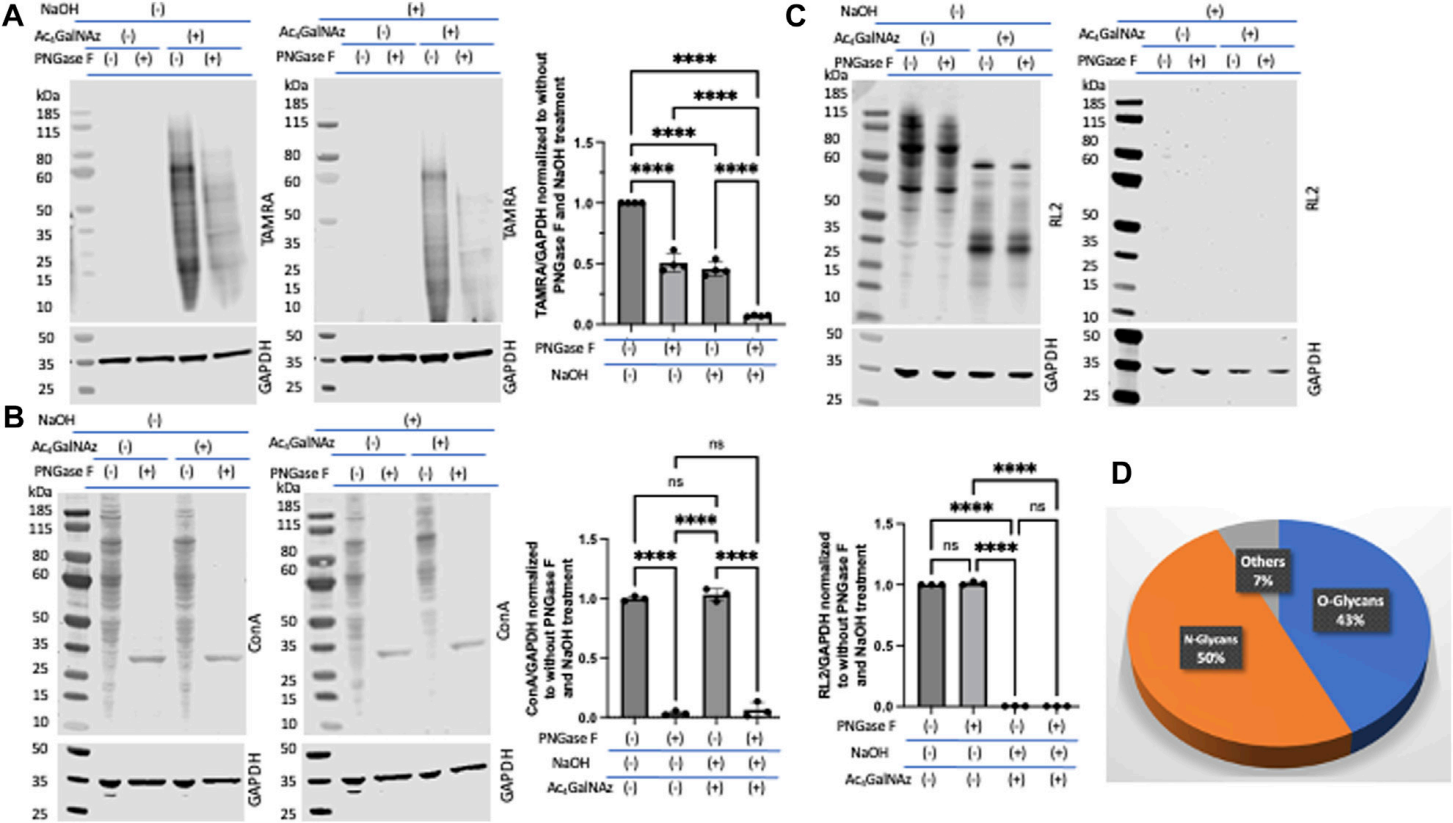
A relatively small portion of Ac_4_GlcNAz labeled beyond *N*- and *O*-glycans. **(A)** Representative blot showing PNGase F and consecutive on-blot β-elimination decreased ∼93% of the Ac_4_GalNAz labeling and graphical quantitation of all replicates (TAMRA signal, N = 4, an ordinary one-way ANOVA test shows ****p < 0.0001). **(B)** Representative blots showing loss of lectin (Con A) binding to endogenous *N*-glycans and graphical quantitation of all replicates (N = 3, an ordinary one-way ANOVA test shows ****p < 0.0001, ns = not significant). **(C)** Representative blot showing loss of *O*-GlcNAc (RL2) signals and graphical quantitation of all replicates (N = 3, an ordinary one-way ANOVA test shows ****p < 0.0001, ns = not significant). **(D)** Relative abundance of different types of glycan labeling by Ac_4_GalNAz.

Due to reports of non-enzymatic incorporation of per-*O*-acetylated MCRs onto proteins (Qin et al., 2018; Qin et al., 2020), we set out to assess relative amounts of azide-derived MCR labeling beyond enzymatic incorporation into *N*- and *O*-glycans. Cells were lysed with detergent to obtain protein and treated with PNGase F, followed by a click reaction to add a TAMRA alkyne. Samples were run out on a gel, transferred to nitrocellulose, and then, subjected to on blot β-elimination conditions under NaOH or H_2_O. The aim was that PNGase F would cleave the *N*-linked glycans and then subsequent β-elimination would remove the glycosidically linked *O*-glycans including glycosaminoglycans (GAGs) (Szekeres et al., 2022) and *S*-glycans (Buchowiecka, 2023), leaving the non-glycosylated protein modifications intact. Here, we found that approximately 7% of the Ac_4_GalNAz signal (detected with the anti-TAMRA antibody) remained (Figures 5A–D). We suspect that this 7% was due to the ability of the per-*O*-acetylated MCR to react with cysteines but remain resistant to β-elimination. This could be because an alkaline solution only cleaves the anomeric center attached through *O*- or *S*-linkages, but not the artificial non-enzymatic *S*-linkages at the C-3, C-4, or C-6 positions generated by the elimination–Michael addition reaction between cysteine thiol and acetates present at those positions of the per-*O*-acetylated MCR.

## Discussion

The use of metabolic chemical reporters has become a widely accessible and easy-to-use approach to study glycoconjugates in a cell or organism. This method allows researchers to utilize probes for imaging, enrichment, profiling, and targeting of glycans (Kiessling and Splain, 2010; Woo et al., 2015; Palaniappan and Bertozzi, 2016; Zheng et al., 2023). Typically, synthetic sugars containing a chemical reporter are given to cells or organisms and are incorporated into glycans by the endogenous biosynthetic machinery. Per-*O*-acetylated sugars are often used to pass the cell membrane and, once inside the cell, are deprotected by non-specific esterases before further processing. Fundamental to understanding the utility of this technique is that monosaccharide precursors are shared by many glycosylation pathways with the interconversion of sugars by epimerases in the cell (Figure 1). In this paper, we proposed a strategy to assess the selectivity of incorporation of an MCR. As a proof-of-concept, we utilized an azide derivative, Ac_4_GalNAz, and alkyne derivatives, Ac_4_GlcNAlk and Ac_4_GalNAlk. Using our strategy (Figure 2), which includes on-blot NaOH, PNGase F, and Endo H treatments, we determined that Ac_4_GalNAz incorporates into ∼50% *N*-glycans and 43% *O*-glycans (Figure 5D). Furthermore, using glycosyltransferase inhibitors, we determined that the majority of the *N*-glycan incorporation was enzymatic and that the majority of *O*-glycan incorporation was enzymatically incorporated by OGT, confirming previous studies (Hang et al., 2003; Boyce et al., 2011). When using the alkyne derivative in HeLa cells, we did not find significant incorporation into *N*-linked glycans or *O*-GlcNAc as has been similarly described by other groups (Batt et al., 2017; Fan et al., 2022). We suggest that the strategy presented in this paper will provide an outline for researchers to help further define and understand their MCR of interest.

Different cell types have different cell-specific glycan signatures (Tao et al., 2008), and it has been previously reported that the MCRs exhibit cell-type-specific metabolism and labeling (Batt et al., 2017). In fact, accumulation of different donor sugars in different cell types correlated with overall labeling (Batt et al., 2017). Keeping this in mind, it is important for researchers to understand the anticipated glycans of the cells being assessed. For example, neuronally derived cells have high levels of gangliosides, and chondrocytes have high levels of GAGs. In these cases, the researcher might consider using *N*-[5-(adamantane-1-yl-methoxy)-pentyl]1-deoxynojirimycin (AMP-DNM) (Bussink et al., 2007) or *p-*nitrophenyl β-D-xyloside (Stevens and Austen, 1982) for the inhibition of ganglioside or GAG synthesis, respectively.

Non-enzymatic or artificial labeling has been described for MCRs as well. The hydrophilic nature of bioorthogonal sugars results in low efficiency of cellular uptake. This hurdle was overcome using per-*O*-acetylated sugars to increase hydrophobicity and membrane permeability. However, per-*O*-acetylated monosaccharides have been shown to chemically react with cysteine residues of intracellular proteins through an elimination–Michael addition reaction between reactive thiols of cysteine and acetate functionality, particularly present at C-3 and C-4 positions on the MCR (Qin et al., 2018; Qin et al., 2020). This non-enzymatic reaction has been termed “artificial cysteine *S*-glyco-modification” (Qin et al., 2020) and could account for significant labeling in cells by the MCR (Qin et al., 2018). In our investigation of the alkyne-derived MCRs, we found that they were not PNGase F cleavable, not enzymatically incorporated by OGT, and only slightly decreased after β-elimination. This could indicate that these MCRs were labeled as artificial *S*-glyco-modifications, possibly due to the concentration used in these studies. Furthermore, difluorinated cyclooctyne (DIFO)-based reagents used for the copper-free click reaction strain-promoted [3 + 2] azide–alkyne cycloaddition (SPAAC) also exhibited non-enzymatic reactivities. These reagents form an adduct with reactive sulfhydryls, which are abundant in the cell. Thus, the use of DIFO-reagents also resulted in high levels of artifactual labeling (Kim et al., 2013). For these reasons, we highlight the use of inhibitors or genetic knockdown of endogenous glycosyltransferases in the cell. Additionally, similar to *O*-linked glycosylation, *S*-linked glycosylation (as opposed to artificial *S*-glyco-modification) would also be subjected to β-elimination. Therefore, the use of these genetic and chemical tools, beyond the on-blot analyses, is essential to assess enzymatic incorporation of the unnatural sugar label.

Despite limitations, the field of bioorthogonal chemistry and the utilization of MCRs have greatly increased our understanding and ability to assess cellular glycans. Employing this approach has allowed the discovery of new classes of glycans such as glycosylated small RNAs (Flynn et al., 2021). Recently, the “bump-and-hole” tactic has been described in which an orthogonal enzyme–sugar pair is engineered (Choi et al., 2019; Schumann et al., 2020; Tian et al., 2021; Cioce et al., 2022; Fan et al., 2022). This adaptation of MCRs allows labeling by individual glycosyltransferases to be selectively interrogated and has been used in a whole organism by genetically encoding a mutant UDP-GlcNAc pyrophosphorylase, AGX2, to specifically metabolize the unnatural 1,3-Pr_2_GlcNAlk sugar. Cell-type-specific expression of the mutant AGX2 allowed for cell-type-specific analysis of MCR incorporation (Fan et al., 2022). This novel approach will promote investigations into the biology of glycan synthesis and enable the development of disease models. Furthermore, these tools have promise as innovative approaches for cancer therapies (Wu D. et al., 2022; Wu S. et al., 2022; Lin et al., 2023) as a drug delivery system (Liu et al., 2019; Yi et al., 2022; Lin et al., 2023) and immunotherapies (Wang et al., 2020), including CAR-T cells (Zhao et al., 2022).

## Materials and methods

### Reagents

All chemicals, reagents, and general laboratory supplies were purchased from Thermo Fisher Scientific or Sigma-Aldrich unless otherwise noted. GlcNAlk was prepared as previously reported (Gurcel et al., 2008; Zaro et al., 2011), and GalNAlk was prepared using the same protocol. Characterization data for GalNAlk matched a previous report (Cheshev et al., 2010). Cell culture reagents including DMEM with 2 mM glutamax were purchased from Gibco. TAMRA alkyne and TAMRA azide (Thermo Fisher Scientific T10183 and T10182, respectively) were dissolved from a stock concentration to 1 mM in DMSO.

Primary antibodies used include those for the following epitopes (catalog number): mouse anti-*O*-GlcNAc (RL2, Thermo Fisher Scientific MA1-072), mouse anti-GAPDH (Abcam ab8245), rabbit anti-TAMRA (Thermo Fisher Scientific, A6397), rabbit anti-GAPDH (Abcam, ab18078), rabbit anti-*O*-GlcNAc transferase (Anti OGT, Santa Cruz Biotechnologies, SC-32921), and biotin-conjugated Con A (Vector Lab, B-1005-5).

Inhibitors were purchased from Sigma-Aldrich with the following specifications: OSMI-1 (Sigma-Aldrich, SML1621) and tunicamycin (Sigma-Aldrich, T7765). Enzymes PNGase F (P0704S) and Endo H (P0702S) were obtained from New England BioLabs.

Primary antibodies were used at 1:1,000 dilution in Odyssey PBS blocking buffer with 0.1% Tween 20. Secondary antibodies including Odyssey IRDye 680 CW goat anti-mouse (Li-COR, 926-68070), IRDye 800 CW goat anti-mouse (Li-COR, 926-32210), IRDye 680 CW goat anti-rabbit (Li-COR, 926-68071), IRDye 800 CW goat anti-rabbit (Li-COR, 926-32211), and IRDye 800 CW streptavidin (Li-COR, 926-32230) were used in 1:10000 dilution in Odyssey PBS blocking buffer containing 0.1% Tween 20.

### Synthesis

**GlcNAlk-Cl**:1,3,4,6-tetra-*O*-acetyl-α/β-D-glucosamine-*N*-pentynoylamide (1.54 g, 3.6 mmol) was treated with 20 mL of acetyl chloride under argon and stirred overnight at room temperature. The reaction was concentrated to dryness on a rotary evaporator, then taken up in toluene, and adsorbed onto Celite. The desired glycosyl chloride was obtained after flash column chromatography purification in hexane:EtOAc using a gradient from 0 to >100% EtOAc (250 mg, 17% yield). ^1^H NMR (CDCl_3_): 6.20 (d, 1H, *J* = 3.8 Hz), 5.94 (d, 1H, *J* = 8.5 Hz), 5.35 (dd, 1H, *J* = 10.6 Hz), 5.23 (t, 1H, *J* = 9.3 Hz), 4.58 (m, 1H), 4.33–4.27 (m, 2H), 4.17–4.10 (m, 2H), 2.53–2.48 (m, 2H), 2.43–2.38 (m, 2H), 2.29 (s, 3H, COC*H*_3_), 2.06 (s, 3H, COC*H*_3_), 2.06 (s, 3H, COC*H*_3_), and 2.01 (t, 1H, *J* = 2.5 Hz).

**Ac**_**3**_ **GlcNAlk-coumarin**:3,4,6-tri-*O*-acetyl-1-chloro-α/β-D-glucosamine-*N*-pentynoylamide (27 mg, 67 μmol) and 7-hydroxycoumarin (10.8 mg, 67 μmol) were combined in dry MeCN under argon. One drop of pyridine was added, and the reaction was stirred overnight and then concentrated under reduced pressure. The desired compound was obtained after preparative HPLC using a gradient of 40%–100% hexane:EtOAc. The relevant fractions were combined and lyophilized to afford 6 mg of a white solid (17% yield). ^1^H NMR (CD_3_OD): 7.90 (dd, 1H, *J* = 9.8, 0.7 Hz), 7.57 (d, 1H, *J* = 8.5 Hz), 7.05 (d, 1H, *J* = 2.3 Hz), 7.02 (dd, 1H, *J* = 8.6, 2.4 Hz), 6.31 (d, 1H, *J* = 9.6 Hz), 5.42 (d, 1H, *J* = 8.3 Hz), 5.37 (dd, 1H, *J* = 10.6, 9.4, Hz), 5.07 (dd, 1H, *J* = 9.9, 8.8 Hz), 4.31 (dd, 1H, *J* = 12.2, 5.5 Hz), 4.23–4.17 (m, 2H), 4.11 (ddd, 1H, *J* = 8.1, 5.9, 2.4 Hz), 2.47–2.42 (m, 2H), 2.37–2.33 (m, 2H), 2.18 (t, 1H, *J* = 2.6 Hz), 2.08 (s, 3H, COC*H*_3_), 2.04 (s, 3H, COC*H*_3_), and 2.02 (s, 3H, COC*H*_3_).

**GlcNAlk-coumarin**: Ac_3_GlcNAlk-coumarin (5 mg, 9.4 μmol) was stirred in a mixture of 1.5 mL MeCN and 2 mL MeOH in an ice bath. Then, 100 μL of aqueous ammonium hydroxide was added, and the reaction was allowed to come to room temperature. After 2 h at RT, the reaction was complete. Concentration to dryness on a rotary evaporator followed by preparative HPLC afforded the desired compound as a white solid (2 mg, 53% yield). ^1^H NMR (DMSO-d_6_): 7.08 (dd, 1H, *J* = 9.5, 0.6 Hz), 6.74 (d, 1H, *J* = 8.3 Hz), 6.23–6.19 (m, 2H), 5.48 (d, 1H, *J* = 9.5 Hz), 4.36 (d, 1H, *J* = 8.3 Hz), 3.18–3.11 (m, 2H), 2.92 (dd, 1H, *J* = 12.3, 5.9 Hz), 2.80 (dd, 1H, *J* = 10.5, 8.7 Hz), 2.73–2.69 (m, 1H), 2.62 (dd, 1H, *J* = 10.0, 8.8 Hz), 1.69–1.58 (m, 4H), and 1.33 (t, 1H, *J* = 2.5 Hz). M + Na for C_20_H_21_NO_8_Na calcd. 426.1165 found 426.1164.

**GalNAlk-coumarin:** GalNAlk-coumarin was synthesized using a similar protocol as used for GlcNAlk-coumarin. M.pt (CH_3_OH) = 193–195°C. ^1^H NMR (CD_3_OD, 600 MHz): 7.92 (d, 1H, *J* = 9.2 Hz), 7.57 (d, 1H, *J* = 9.2 Hz), 7.09–7.04 (m, 2 H), 6.31 (d, 1H, *J* = 9.2 Hz), 5.17 (d, 1H, *J* = 9.2 Hz, *H*-1), 4.28 (dd, 1H, *J* = 8.7, 10.2 Hz, *H*-2), 3.95 (d, 1H, *J* = 3.9 Hz, *H*-4), 3.88–3.74 (m, 4H, *H*-3, *H*-6, *H-*6′, -N*H*), 2.53–2.43 (m, 4H), 2.16 (brs, 1H); ^13^C NMR (CD_3_OD, 125 MHz): 175.7 (*C*=O), 163.9 (*C*=O), 162.9, 157.5, 146.4, 131.2, 116.3, 115.2, 105.7, 101.6 (*C*-1), 78.2 (*C*-3/*C*-5), 73.6 (*C*-5/*C*-3), 71.1 (alkyn-*C*), 70.6 (*C*-4), 63.3 (*C*-6), 54.8 (*C*-2), 50.4, 37.3, and 16.5. M + Na for C_20_H_21_NO_8_Na calcd. 426.1165 found 426.1167.

**GlcNAc-coumarin**: ^1^H NMR (CD_3_OD): 7.89 (d, 1H, *J* = 9.6 Hz), 7.55 (d, 1H, *J* = 8.5 Hz), 7.03–6.99 (m, 2H), 6.29 (d, 1H, *J* = 9.5 Hz), 5.18 (d, 1H, *J* = 8.4 Hz), 3.97–3.91 (m, 2H), 3.72 (dd, 1H, *J* = 12.0, 5.6 Hz), 3.59 (dd, 1H, *J* = 10.4, 8.7 Hz), 3.54–3.40 (m, 2H), and 1.98 (s, 3H). M + Na for C_17_H_19_NO_8_Na calcd. 388.1008 found 388.1006.

**GalNAc-coumarin:** M.pt (CH_3_OH) = 172–174°C. ^1^H NMR (CD_3_OD): 7.89 (d, 1 H, *J* = 9.7 Hz); 7.55 (d, 1 H, *J* = 8.5 Hz); 7.00–7.04 (m, 2 H); 6.28 (d, 1H, *J* = 9.6 Hz); 5.16 (d, 1 H, *J* = 8.3 Hz); 4.23 (dd, 1 H, *J* = 8.3, 10.7 Hz); 3.92 (d, 1 H, *J* = 3.2 Hz); 3.72–3.84 (m, 4 H); and 1.98 (s, 3 H). M + Na for C_17_H_19_NO_8_Na calcd. 388.1008 found 388.1004.

### Cell culture

HeLa cells were cultured in DMEM supplemented with 10% FBS, penicillin (100 U/mL), and streptomycin (P/S, 1 mg/mL) at 37 °C in a humidified incubator with 5% CO_2_. For experiments, cells were seeded at a density of 100k cells/well in a six-well tissue culture plate and 500k cells in a 10 cm plate. Cells were allowed to adhere overnight, and then, an appropriate amount of acetylated sugar was added for the desired final concentration from a 100 mM stock. Alternatively, the equivalent volume of DMSO was added as a negative control. After the appropriate time, cells were isolated by trypsinization, counted, and centrifuged at 1,000 rpm for 5 min in 15 mL conical tubes. Cell pellets were stored at -20 °C for temporary storage or -80 °C for longer storage until use.

### Immunoblot

Cell pellets were lysed with RIPA lysis buffer on ice for 10 min with occasional shaking and then centrifuged at 4 °C at maximum speed for 10 min in 1.5 mL Eppendorf tubes. Cell lysates were stored at -20 °C for temporary storage or -80 °C for longer storage. Protein concentration was determined by BCA assay (Pierce, Thermo Fisher Scientific) and normalized to the lowest concentration using RIPA buffer. For CuAAC labeling using the Click-iT kit C10276, to 25 μL of lysate (up to 50 μg protein), 100 μL buffer A (containing 40 μM of TAMRA azide), 10 μL CuSO_4_, 10 μL additive 1, and after 2 min, 20 μL additive 2 were added. The samples were mixed for 25 min after which the protein was precipitated with methanol/chloroform. For MeOH/CHCl_3_, 600 μL MeOH, 150 μL CHCl_3_, and 400 μL water were added, and the samples were mixed. The samples were centrifuged for 5 min at maximum speed and the top, aqueous layer (upper colorless) was removed and discarded. Next, 450 μL MeOH was added in two separate washes after which the samples were centrifuged at maximum speed, and the protein resided at the bottom of the tube. The resulting protein pellet was air-dried (0.5–1 h). Last, protein was resuspended in an appropriate volume of LDS dye with BME. Protein was resolved by SDS-PAGE: 4%–12% Bis-tris gels (Invitrogen) were used with MOPS to resolve proteins after which they were transferred to a 0.2 μm nitrocellulose membrane using the Invitrogen™ iBlot™ 2 Gel Transfer Device (IB21001). The blots were then blocked with Odyssey PBS blocking buffer at room temperature for 1 h and then incubated with the appropriate primary antibody (1:1,000 dilution) in Odyssey PBS blocking buffer with 0.1% Tween 20 overnight at 4 °C. The next day, the blots were washed three times in 1XPBSTw for 10 min each and incubated with the appropriate secondary Odyssey antibodies (1:10000 dilution) in Odyssey PBS blocking buffer with 0.1% Tween 20 at room temperature for 1 h. The blots were then washed thrice with 1XPBSTw and developed under the Odyssey instrument. The blots were quantified using Image Studio software (Li-Cor). A fixed rectangular box was drawn around each lane measuring median intensities with local background subtraction.

### β-Elimination

Cells were treated with 100 μM of the proper MCR or equivalent amount of DMSO as a negative control and incubated for 48 h. Then, cells were collected by trypsinization and pelleted by centrifugation for 5 min at 1,000 *rpm*. Following washing twice with ice-cold 1XPBS (1 mL), cell extracts were collected using RIPA buffer. Protein concentration was determined by BCA assay (Pierce, Thermo Fisher Scientific) and normalized to the lowest protein concentration using RIPA buffer. Both DMSO- and sample-treated cell lysates were subjected to CuAAC reaction with TAMRA alkyne/azide and lysates, run on 10 well gels, and transferred onto nitrocellulose membranes as described earlier. After a 1XPBS wash for 10 min, the membrane was cut in half, and one- half was incubated with 55 mM NaOH and the other with water for 24 h at 40 °C. The blots were then washed thrice with 1XPBSTw and then blocked with Odyssey PBS blocking buffer at room temperature for 1 h. The blots were then incubated with the appropriate primary antibody in Odyssey PBS blocking buffer with 0.1% Tween 20 overnight at 4 °C. Both the anti-RL2 antibody (Thermo Fisher Scientific, MA1-072), anti-TAMRA antibody (Thermo Fisher Scientific, A6397), and anti-GAPDH (Abcam, ab8245 or Abcam, ab18078) as loading control were used at a 1:1,000 dilution. The blots were then washed three times in 1XPBSTw for 10 min each, incubated with the appropriate secondary Odyssey antibodies (1:10000 dilution) in Odyssey PBS blocking buffer with 0.1% Tween 20 at room temperature for 1 h, washed thrice with 1XPBSTw, and developed under the Odyssey instrument simultaneously. The blots were quantified simultaneously using Image Studio software (Li-Cor). A fixed rectangular box was drawn around each lane or band measuring median intensities with local background subtraction.

### PNGase F and Endo H treatment

PNGase F (P0704S) and Endo H (P0702S) were obtained from New England BioLabs, and treatments were performed according to the manufacturer’s protocol with some changes as described below. Cells were treated with 100 μM of the proper MCR or equivalent amount of DMSO as a negative control and incubated for 48 h. Then, cells were collected by trypsinization or scraping and pelleted by centrifugation for 5 min at 1,000 *rpm*. Following washing twice with ice-cold 1XPBS (1 mL), cell extracts were collected using RIPA buffer. Protein concentration was determined by BCA assay (Pierce, Thermo Fisher Scientific) and diluted to 1 mg/mL using RIPA buffer. Both the DMSO-treated and sample-treated cell lysates were subjected to enzyme treatment and water treatment as negative control. For PNGase F assay, to 10 μg of protein, 1 μL of 10×glycoprotein denaturing buffer was added and incubated for 10 min at 100 °C. Then, 2 μL of GlycoBuffer 2, 2 μL of 10% NP-40, 1 μL of PNGase F, and 4 μL of deionized water (to make final volume of 20 mL) were added and incubated for 1 h at 37 °C. For Endo H assay, to the 10 μg of protein, 1 μL of 10×glycoprotein denaturing buffer was added and incubated for 10 min at 100 °C. Then, 2 μL of 10×GlycoBuffer 3, 1 μL of Endo H, and 6 mL of deionized water (to make a final volume of 20 mL) were added and incubated for 1 h at 37 °C. Enzyme (PNGase F and Endo H)-treated and water-treated cell lysates were separately subjected to CuAAC reaction with TAMRA alkyne/azide. They were subjected to immunoblotting on the nitrocellulose membrane, and the blots were blocked with Odyssey PBS blocking buffer at room temperature for 1 h. The blots were then incubated with the appropriate primary antibody in Odyssey PBS blocking buffer with 0.1% Tween 20 overnight at 4 °C. Both the anti-GAPDH (Abcam ab8245) and anti-TAMRA antibodies (Thermo Fisher Scientific, A6397) were used at a 1:1,000 dilution. The blots were then washed three times in 1XPBSTw for 10 min each, incubated with the appropriate secondary Odyssey antibodies (1:10000 dilution) in Odyssey PBS blocking buffer with 0.1% Tween 20 at room temperature for 1 h, washed thrice with 1XPBSTw, and developed under the Odyssey instrument. Then, the blots were stripped using stripping buffer at 4 °C overnight, washed, and blocked with Odyssey PBS blocking buffer at room temperature for 1 h. The blots were then incubated with biotin-conjugated Concanavalin A (Vector Lab, B-1005-5) and diluted 1:1,000 in Odyssey PBS blocking buffer with 0.1% Tween 20 for 1 h. The blot was then washed thrice with 1XPBSTw for 10 min each. Next, the blot was incubated with IRDye 800 CW streptavidin Odyssey secondary antibody at 1:10000 in Odyssey PBS blocking buffer with 0.1% Tween 20 for 1 h. After being washed thrice with 1XPBSTw for 10 min each, the blot was developed using the Odyssey instrument. The blots were quantified using Image Studio software (Li-Cor). A fixed rectangular box was drawn around each lane or band, measuring median intensities with local background subtraction.

### Inhibitor study

HeLa cells were cultured as described earlier. For inhibitor studies, different concentrations of the inhibitors were added after 16 h from the initial cell seeding and incubated for another 12 h. Cells were treated with 100 μM of the proper MCR and incubated for an additional 48 h. Cell lysate was collected for immunoblotting as previously described.

### siRNA

HeLa cells were cultured as described above. For siRNA experiment, DMEM (Gibco, A14430 without phenol red) was supplemented as mentioned above with or without antibiotics. The following siRNA reagents were purchased from Santa Cruz: transfection reagent (sc-29528), NCOAT siRNA construct (human) (sc-62667), OGT siRNA construct (human) (sc-40780), and Control siRNA-A (sc-37007). Lyophilized siRNA duplex was resuspended in 330 μL (NCOAT and OGT) or 66 μL (controls) of the RNAse-free water provided. This yields a 10 μM solution in 10 μM Tris-HCl pH 8.0, 20 mM NaCl, and 1 mM EDTA. The samples were aliquoted to 15 μL each and stored at -20 °C. HeLa cells were grown in DMEM until experiment was planned. HeLa cells were seeded in six-well plates in a normal growth medium without P/S and grown to ∼50% confluency in an incubator at 37°C, 5% CO_2_. On day 1, for transfections, we diluted 4 μL siRNA duplex (i.e., 0.25–1 μg or 20–80 pmols siRNA) into 100 μL PBS. Likewise, we diluted 4 μL siRNA transfection reagent in 100 μL PBS, added equal volumes of siRNA duplex and diluted transfection reagent and mixed gently, and incubated at room temperature for 20 min, and 800 μL PBS was added to each tube containing the transfection mixture. After washing cells with 1xPBS and aspirating out the liquid, the duplex/transfection reagent mixture was added to the wells, and cells were incubated for 5 h at 37 °C in the 5% CO_2_ incubator. After the incubation period, 1 mL normal growth medium was added, and the cells were incubated overnight at 37 °C.

On day 2, day 1’s procedure was repeated with one exception: when adding in the normal growth medium, DMSO or 100 μM Ac_4_GlcNAlk was added to half of the cells. After 24 h of incubation, the cells were isolated by trypsinization and processed as described for immunoblot above.

### Purification of his-tagged BtGH84

Rosetta 2 (DE3) (Novagen) cells were transformed and grown on LB plates with 34 μg/ml chloramphenicol and 20 μg/ml kanamycin. Approximately three colonies were combined and grown overnight at 37 °C in a 20 mL starter culture of LB with CAM/KAN as mentioned above. The next day, the culture was expanded 1:30 with LB/CAM/KAN and grown at 37 °C to OD-600 0.88, induced with 1 mM IPTG, and grown 4.5 h. Cultures were centrifuged at 6k xg at 4 °C and pellets frozen at -80 °C. The pellets were thawed and resuspended in 100 mM Tris-HCl (pH 8.0), 100 mM NaCl, and 1 mg/ml lysozyme, then sonicated, and centrifuged at 10k xg for 25 min at 4°C. 2 mM DTT and 15 mM imidazole (pH 8.0) were added to cleared lysate, which was applied to 2 mL NiNTA agarose (Qiagen) that had been previously washed with 100 mM Tris-HCl and 100 mM NaCl and equilibrated with lysis buffer plus 15 mM imidazole. Binding was performed at 4 °C for 2 h rotating end-over-end. Resin was pelleted at 1k xg for 1 min, flow through removed, and washed twice with 20 mL of 100 mM Tris-HCl (pH 7.4), 100 mM NaCl, and 15 mM imidazole. The resin was then transferred to a BioRad glass column and further washed with approx. 20 CVs of wash buffer. Protein was eluted six times (∼500 μL each) with 20 mM Tris-HCl (pH 7.4), 500 mM NaCl, 250 mM imidazole (pH 8.0), and 10% glycerol. Elutions two to five were combined for a total volume of 2.5 mL, applied to a PD-10 desalting column equilibrated with PBS, and then, eluted with 3.5 mL PBS and collected in five separate aliquots. An additional 750 μL PBS was applied to the column for a sixth elution aliquot. Elutions one to five were combined after monitoring by Coomassie. Elutions were then aliquoted, frozen on dry ice, and stored at -80 °C.

Mutations were made on the BtGH84-wt pET28a template using the primers indicated in the Supplementary Material with a QuikChange II kit (Agilent). All mutations were confirmed by sequencing using either the pET28a T7 promoter primer or an internal BtGH84 primer.

Proteins were expressed and purified as described earlier for WT, except on a smaller scale. A 35 mL culture was induced, pelleted, and lysed with 3 mL lysozyme buffer containing 100 mM Tris-HCl pH 8.0, 100 mM NaCl, and 2 mg/ml lysosome. Following lysozyme digest, 1 mM DTT and 15 mM imidazole were added, and NaCl was adjusted to 500 mM. Lysates were pulled through a 19-gauge syringe 12 x (instead of sonicated) and cleared at 13k xg for 20 min at 4 °C. Cleared lysates were applied to 300 μL of NiNTA equilibrated with lysis buffer. The samples were rotated at 4 °C for 1 h; then, resin was washed twice with 4 mL wash buffer and loaded into BioRad micro-Bio-Spin Columns (#732-6204). The resin was further washed with two CVs of wash buffer, and proteins were released with one 500 μL bolus of elution buffer, which was allowed to drip via gravity for approximately 10 min prior to centrifugation at 100 *g* for 30 s. Buffer exchange to PBS was performed on a mini–PD MiniTrap G-25 desalting column (GE 28-9180-07, same as PD-10 but smaller).

### *In vitro O*-GlcNAc cleavage with BtGH84

HeLa cell lysates treated with or without alkynyl sugars were collected and subjected to CuAAC reaction with TAMRA azide followed by treatment with or without BtGH84 in solution. For the *in vitro* reaction, 10 μg of protein was diluted in BtGH84 reaction buffer (50 mM sodium phosphate, 1% NP-40, 500 mM NaCl, and 5 mM BME with HALT protease inhibitor) and incubated with 60 μg of BtGH84 or water in 600 μL of buffer total. The reaction was incubated at 37 °C overnight after which the proteins were precipitated with MeOH/CHCl_3_, resuspended in LDS dye with BME, boiled, and resolved by SDS-PAGE. The samples were transferred to nitrocellulose as described earlier after which they were blocked with Odyssey blocking buffer and probed for *O*-GlcNAc and TAMRA as described above.

For initial reactions to determine which mutants would cleave the unnatural *O*-GlcNAlk, assays with coumarin-GlcNAlk were performed in triplicate. Enzymes were diluted to 100 μg/mL (50 mM phosphate buffer, pH 6, 0.1% NP-40). To each reaction, 100 ng of enzyme was added to 39 μL of buffer and 10 μL 20 μM coumarin-GlcNAc or coumarin-GlcNAlk. The reaction proceeded for 10 min at 37°C, and the reaction was quenched with 150 μL 0.5 M Na_2_CO_3_. The progress of the enzyme reaction was determined by measuring the extent of umbelliferon liberated as determined by fluorescence measurements using a Polarstar Omega and comparison to a standard curve of coumarin under identical buffer conditions. Each fluorescence background at different concentrations of the substrate without enzyme was subtracted from the enzymatic reactions at its corresponding substrate concentration, and anything resulting in a negative number is reflected as 0.

### Statistics

GraphPad (version 10) prism was used for all statistics. A two-way ANOVA was used to determine significance as indicated in figure legends. *p*-values less than 0.05 were considered statistically significant.

## Data Availability

The original contributions presented in the study are included in the article/Supplementary Material; further inquiries can be directed to the corresponding author.

## Supplementary material

SUPPLEMENTARY FIGURE S1 Decision tree outlining a proposed strategy to determine the type of labeling by MCRs. Nucleotide sugars and glycolipids are organic solvent (MeOH, acetone) and/or detergent (Triton X100) soluble and, hence, washed off during permeabilization or extraction using either of the two reagents. Nucleotide sugars can be detected on TLC using H_2_SO_4_ charring, and glycolipids can be detected using both H_2_SO_4_ and *p*-anisaldehyde charring. Both mucin-type and intracellular *O*-GlcNAcylation are susceptible to an alkaline medium through β-elimination, whereas *N*-glycans or other non-enzymatic artificial *S*-modification (linkage of cysteine residue to the C-3, C-4, or C-6 hydroxyl group in the sugar residue) are not. Inhibitors against the enzymes for intercellular *O*-GlcNAc cycling OSMI-1 and/or TMG alter the *O*-GlcNAcylation levels and have no direct impact on the mucin-type *O*-linked glycan level. PNGase F cleaves all three types of *N*-glycans and releases the oligosaccharide from the asparagine residue, whereas Endo H cleaves between the two *N*-acetyl-D-glucosamine residues in the chitobiose core for oligomannose and hybrid *N*-glycans but not complex *N*-glycans. α-Mannosidase I (ER mannosidase) inhibitors (1-deoxymannojirimycin and kifunensine) impede the formation of hybrid *N*-glycan by occluding the trimming of the oligomannose structure to the downstream Man_5_GlcNAc_2_Asn residue required for hybrid *N*-glycan formation.

SUPPLEMENTARY FIGURE S2 Assessment of *O*-linked glycans from cells treated with alkyne-modified MCRs. **(A)** Blot showing that coupled with thiamet G (TMG) treatment, β-elimination of *O*-GlcNAc (using RL2 antibody) was complete, whereas the alkyne-derived signal slightly decreased. **(B)** Blot showing siRNA of OGT reduced OGT and *O*-GlcNAc levels, but the Ac_4_GlcNAlk-derived signal remained. Membranes were cut to allow analysis using indicated antibodies.

SUPPLEMENTARY FIGURE S3
*In vitro* glycanase assay to screen BtGH84 mutants. **(A)** BtGH84 was expressed in *E. coli* and purified by NiNTA agarose. Representative gel for purification of WT BtGH84. **(B)** Fluorescent signal derived from coumarin-sugar cleavage by WT BtGH84 and mutants. Only the C277A mutant had the ability to cleave the coumarin-alkyne. **(C)** Lysates were treated with BtGH84-WT or C277A and then divided for analysis of *O*-GlcNAc and their respective alkyne-derived signal following CuAAc reaction.

SUPPLEMENTARY FIGURE S4 Portion of *N*-linked glycans labeled by alkyne-derived MCRs lysates treated with **(A)** PNGase F or **(B)** Endo H did not greatly affect the alkyne-derived signal. L-PHA and Con A lectins were used to confirm the activity of the enzymes.

SUPPLEMENTARY FIGURE S5 Portion of *N*-linked glycans labeled by Ac_4_GalNAz with cells collected by scrapping. **(A)** Representative blot showing PNGase F treatment significantly diminished Ac_4_GalNAz labeling similar to cells collected by trypsinization (Figure 4) and graphical quantitation of replicates (TAMRA signal, N = 4, an ordinary one-way ANOVA test shows ****P <0.0001, ns = Not significant). **(B)** Representative blot indicating that endogenous lectin labeling was removed by PNGase F treatment and graphical quantitation of all replicates (Con A signal, N = 4, an ordinary one-way ANOVA test shows ****P <0.0001).

SUPPLEMENTARY TABLE S1 Primers used for mutation of WT BtGH84 pET28a plasmid.
