# Supplementary figures and images for: Tools and tactics to define specificity of metabolic chemical reporters
Source: Front Mol Biosci. 2023 Dec 7;10:1286690. doi: 10.3389/fmolb.2023.1286690 (PMC10740162; doi:10.3389/fmolb.2023.1286690)

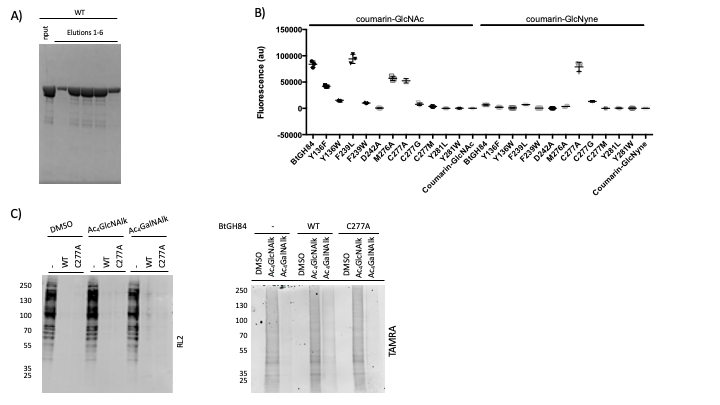

Supplement: Supplementary file 1 [file Image3.TIFF]

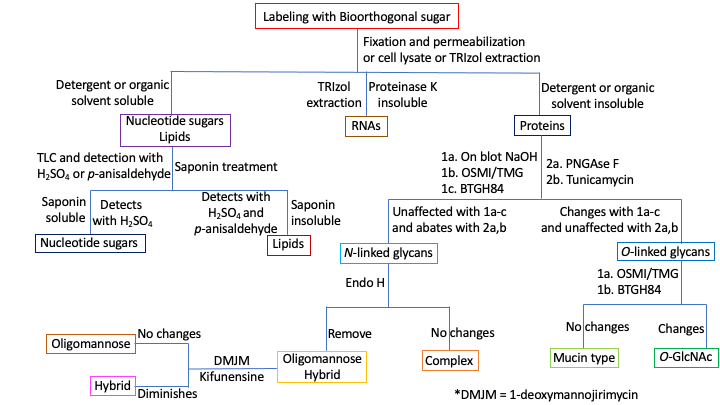

Supplement: Supplementary file 2 [file Image1.TIFF]

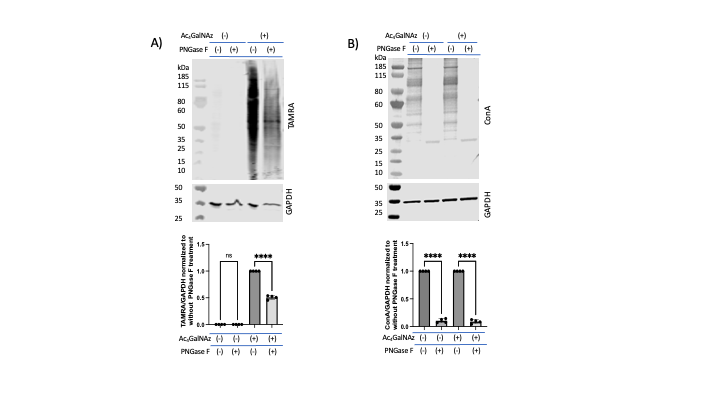

Supplement: Supplementary file 3 [file Image5.TIFF]

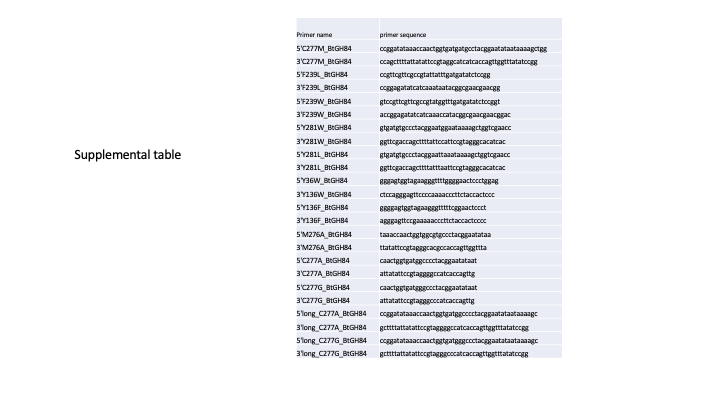

Supplement: Supplementary file 4 [file Image6.TIFF]

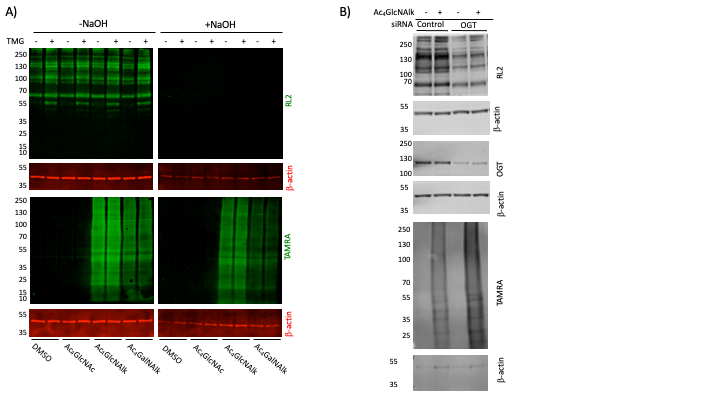

Supplement: Supplementary file 5 [file Image2.TIFF]

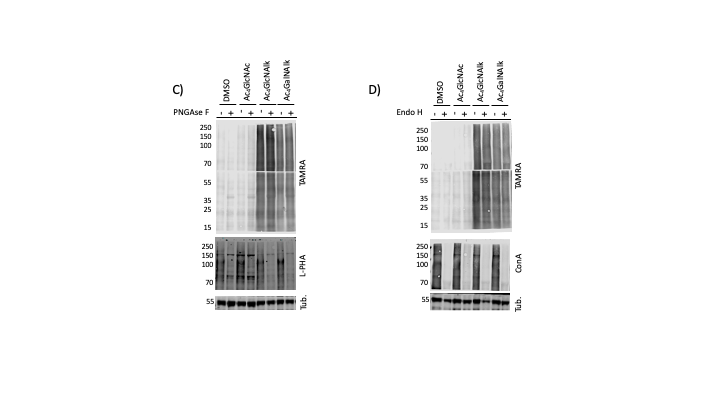

Supplement: Supplementary file 6 [file Image4.TIFF]
